# Certificated Blind Masseurs

**Published:** 1923-04

**Authors:** Robert Jones

**Affiliations:** 11 Nelson Street, Liverpool


### Certificated Blind Masseurs.

Sir,—I am much interested in the work of the Association of Certificated Blind Masseurs, of which I have the pleasure to be President, and I should be very grateful if you could find space in your valuable journal to publish this letter.

The Association has recently published leaflets showing the locations of its members in London and all parts of the British Isles. May I venture to suggest that all registered medical practitioners would do well to obtain copies from the Secretary of the Association, 224-6-8 Great Portland Street, London, W.1. ? These leaflets show that members of the Association, both masseurs and masseuses, are established in practically all the large towns, as well as in all parts of London and the suburbs. As massage is work which it is generally recognised can be most efficiently carried out by properly trained blind people, those doctors who can see their way to the employment of the members of the Association of Certificated Blind Masseurs will be aiding in a work of national importance.

Robert Jones.

11 Nelson Street, Liverpool.

### PRESENTATIONS TO HOSPITAL ORGANISERS.

THERE have recently been three presentations to hospital secretaries and organisers, all of whom have done valuable work for the institutions with which they are connected. Mr. Walter Wormald has been presented with a tea service in recognition of his twenty-five years work as secretary of the Leeds Workpeople's Hospital Fund. Mr. H. M. Howard, the honorary secretary of the West Norfolk and Lynn Hospital, has been presented with a silver inkstand and a pair of silver salt cellars in recognition of his good work during the past twelve years. Mr. Howard has taken great interest in the village contribution scheme of the district and in the Hospital Sunday Fund movement. A gold watch has been presented to Mr. William R. Wilson by the Workmen's Committee of Durham County Hospital and the subscribers on his completion of twenty-one years work in the post of secretary. The length of service given by hospital men is often remarkable, and the changes that have taken place during Mr. Wilson's administration concern not merely the equipment and management of voluntary hospitals, nor the wonderful growth of working-men's support, but the improved status of the hospital profession.

